# Causal relationship of CA3 back-projection to the dentate gyrus and its role in CA1 fast ripple generation

**DOI:** 10.1186/s12868-021-00641-4

**Published:** 2021-05-17

**Authors:** Miguel A. Núñez-Ochoa, Gustavo A. Chiprés-Tinajero, Nadia P. González-Domínguez, Laura Medina-Ceja

**Affiliations:** 1 Laboratory of Neurophysiology, Department of Cellular and Molecular Biology, CUCBA, University of Guadalajara, Camino Ing. R. Padilla Sánchez 2100, Las Agujas, Nextipac, CP 45110 Zapopan, Jalisco Mexico; 2 Biomedical Sciences, CUCS, University of Guadalajara, Sierra Mojada 950, Colonia Independencia, CP 44340 Guadalajara, Jalisco Mexico

**Keywords:** Fast ripples, Granger causality, Hippocampus, In vivo studies, Pilocarpine model, Theta rhythm

## Abstract

**Background:**

Pathophysiological evidence from temporal lobe epilepsy models highlights the hippocampus as the most affected structure due to its high degree of neuroplasticity and control of the dynamics of limbic structures, which are necessary to encode information, conferring to it an intrinsic epileptogenicity. A loss in this control results in observable oscillatory perturbations called fast ripples, in epileptic rats those events are found in CA1, CA3, and the dentate gyrus (DG), which are the principal regions of the trisynaptic circuit of the hippocampus. The present work used Granger causality to address which relationships among these three regions of the trisynaptic circuit are needed to cause fast ripples in CA1 in an in vivo model. For these purposes, male Wistar rats (210–300 g) were injected with a single dose of pilocarpine hydrochloride (2.4 mg/2 µl) into the right lateral ventricle and video-monitored 24 h/day to detect spontaneous and recurrent seizures. Once detected, rats were implanted with microelectrodes in these regions (fixed-recording tungsten wire electrodes, 60-μm outer diameter) ipsilateral to the pilocarpine injection. A total of 336 fast ripples were recorded and probabilistically characterized, from those fast ripples we made a subset of all the fast ripple events associated with sharp-waves in CA1 region (n = 40) to analyze them with Granger Causality.

**Results:**

Our results support existing evidence in vitro in which fast ripple events in CA1 are initiated by CA3 multiunit activity and describe a general synchronization in the theta band across the three regions analyzed DG, CA3, and CA1, just before the fast ripple event in CA1 have begun.

**Conclusion:**

This in vivo study highlights the causal participation of the CA3 back-projection to the DG, a connection commonly overlooked in the trisynaptic circuit, as a facilitator of a closed-loop among these regions that prolongs the excitatory activity of CA3. We speculate that the loss of inhibitory drive of DG and the mechanisms of ripple-related memory consolidation in which also the CA3 back-projection to DG has a fundamental role might be underlying processes of the fast ripples generation in CA1.

**Supplementary Information:**

The online version contains supplementary material available at 10.1186/s12868-021-00641-4.

## Background

Temporal Lobe Epilepsy (TLE) is the main form of localized epilepsy, a highly drug-resistant disease that is generally treated using surgical intervention [1, 2], being hippocampal sclerosis the most frequent pathophysiological evidence. This makes the hippocampal region of special interest to investigate the mechanisms related to the development and establishment of the disease [3–6].

Hippocampus exhibits a high degree of neuroplasticity and great control of the temporal dynamics of its circuits to encode information [7–11], this property confers an intrinsic epileptogenicity because the loss of excitatory/inhibitory control [12–14] might be fixed as a stable state in the main circuit of the structure, the trisynaptic circuit [10, 15–17].

There are several proposals of the mechanisms in the trisynaptic circuit that generate and underlie fast ripple activity [18–27], which is an activity observed before and during seizures [28–30] in the range of 250–600 Hz, commonly recorded in quiet wakefulness or slow-wave sleep states and related to clusters of pathologically associated neurons [19, 28]. This activity is considered as a biomarker of epileptogenic processes [31–34] and has been described to be produced by neuronal populations ascribed to a volume of approximately 1 mm^3^ of tissue [20, 24]. Due to its area specificity and that are commonly found in seizure onset zone, fast ripple activity has been used as a surgical reference to detect candidate areas for resection, with resulting seizure-free outcomes in patients [35–37].

Although there is evidence around the changes needed in the neural circuits to develop fast ripple events, only a few of those hypothesis has been tested in an in vivo model, therefore the present work aims to describe the causal dynamics needed in the trisynaptic circuit to develop fast ripples in the CA1 region, and its relationship with the temporal dynamics of neuronal firing and frequency synchronization among the three regions analyzed: the dentate gyrus (DG), CA3 and CA1, using an in vivo pilocarpine model of TLE.

Our results support existing evidence that fast ripple activity begins in the CA3 region with theta synchronization across regions and shows the participation of the CA3 back-projection to DG in the FR generating process in vivo. Moreover, adding evidence and linking the fact that fast ripples could be a consequence of the same mechanisms used by memory consolidation associated with ripples. These mechanisms are potentiated by both the recurrent excitation of CA3 and the loss of inhibitory control exerted by the DG in the hippocampus, in which that connection has a fundamental role.

## Methods

### Temporal lobe epilepsy model using pilocarpine administration

Adult male Wistar rats (210–300 g; source of animals: Institute of Neurobiology, UNAM) were used for this study in a manner designed to minimize the number of animals used and suffering. Animals were housed in a temperature-regulated room (22 ± 2 °C), on a 12-h day/12-h night cycle (lights on from 7:00 a.m. to 7:00 p.m.) and had free access to food and water. This protocol conformed to the Rules for Research in Health Matters (Mexican Official Norms NOM-062-ZOO-1999, NOM-033-ZOO-1995), with the approval of the local Animal Care Committee of University Center for Biological and Agricultural Sciences from University of Guadalajara.

TLE model induced by pilocarpine was taken from [38]. Rats were anaesthetized with oxygen-isoflurane prior to pilocarpine injection into the right lateral ventricle of the brain (AP: − 4.5 mm, ML: − 5.2 mm and DV: − 7 mm) with a single dose of pilocarpine hydrochloride (2.4 mg/2 μl); Sigma-Aldrich, USA) using a needle connected to an injection pump attached to the stereotaxic framework (Stoelting Co., IL, USA). The animals were observed and scored according to the Racine scale [39]. *Status epilepticus* (scale 4 and 5) induced in these rats was stopped via an injection of diazepam (5–10 mg/kg, i.p.) to increase the survival of animals; a second application of diazepam was administered if necessary. These animals were video-monitored 24 h/day; only animals at which were detected spontaneous and recurrent seizures were used in this study; we defined as the first spontaneous seizure when has a behavioral severity higher than 4 in the Racine scale which is a tonic–clonic seizure.

### Microelectrode implantation surgery

Rats with spontaneous and recurrent seizures were eligible for microelectrode implantation 15 days after the first spontaneous seizure was detected (n = 6). For implantation, rats were oxygen-isoflurane anesthetized and fixed to a stereotactic frame. Fixed-recording tungsten wire electrodes (60-μm outer diameter), consisting of pairs with a 500-µm vertical tip separation, were implanted into the dentate gyrus region (DG, AP: − 6.48 mm, ML: − 4.6 mm and DV: − 6 mm from bregma), CA3 region (AP: − 5.04 mm, ML: − 4.5 mm and: − 6.5 mm from bregma), and CA1 region (AP: − 6.72 mm, ML: − 5.8 mm and DV: − 5.3 mm from bregma). All microelectrodes were implanted ipsilateral to the pilocarpine injection. Two stainless steel screws were driven into the bone above the bregma, which served as indifferent and ground electrodes. After surgery, rats were treated with enrofloxacin (Enroxil, 22 mg/kg weight, oral administration) and paracetamol (700 mg/kg weight, oral administration) to prevent pain and infections.

### EEG recordings

After 3 days of recovery from the microelectrode implantation, animals were recorded in free-movement conditions for 60 min/day on days 19, 20, 21, 25, and 32 (5 days) after the first spontaneous seizure. The recordings were performed in a polygraph (Model 7D, Grass Technologies, RI, USA) at a bandwidth of 0.1 to 5 kHz, sampling at 5 kHz per channel (3 channels) with 12-bit precision using an iMac A1048 (Apple, USA) and MP150 (BIOPAC Systems, CA, USA) as an analog to digital converter, and AcqKnowledge Data Acquisition software 4.0 as the user interface (BIOPAC Systems, USA).

### Fast ripple detection

Following criteria were used for fast ripples selection: (a) EEG recordings were visually inspected for fast ripples candidates; (b) from these selected recordings with fast ripple candidates, recordings with a line-noise > 15 μV or a peak-to-peak amplitude greater than > 150 μV were eliminated to prevent false positives by signal/noise ratio, and due artifacts by movement, respectively; and (c) the remaining recordings underwent a continuous wavelet transformation to guarantee that the frequency event was temporarily delimited, and there were no harmonics of 60 Hz in the signal. This analysis was bound to the band of 250 Hz at 600 Hz and normalized to the highest wavelet power in the fast ripple bandwidth between the different recording channels. If the frequency profile of the analyzed potential fast ripple event coincided with the expected (power in the fast ripple band co-localized with the potential fast ripples by visual inspection), then the recording was classified as a fast ripple.

Fast ripple events that met the classification criteria were filtered in the band of 250 to 600 Hz using a forward–backward zero-phase FIR filter with 512 order, then evaluated. Parameters such as peak-to-peak amplitude; power frequency, defined as the highest peak in power spectral density; power, defined as the mean sum of squares of the power frequency; and duration of the event were analyzed for the different recording regions. Causal dynamics and temporal and frequency relationships in the trisynaptic circuit before and after a fast ripple event were described. All analyses were performed offline with custom programs written in MATLAB (MathWorks Inc., USA), Python (Python Software Foundation License, USA), or R (R Foundation for Statistical Computing, GNU General Public License, USA).

There were recorded 336 fast ripple events (n = 6 rats) mainly in rats on quiet wakefulness, distributed per region as follows, 75 in DG, 71 in CA3 and 190 in CA1. A subset of 40 sharp-wave associated CA1 fast ripple events (n = 6 rats) were used to conduct the multiunit activity, wavelet coherence and Granger causality analyses; to selected only sharp-wave associated fast ripples we measured the duration of the putative sharp-waves, and only events greater than 70 ms [40] were selected (168 ± 69 ms). The main reasons to select that subset were: (1) when a fast ripple event was associated with a sharp wave, the probability of a false-positive result by visual inspection was lower. This is because the slow component of the sharp-wave made it easier to visually identify high-frequency oscillations; (2) the hypothesis that fast ripples were associated with sharp waves, observed in the same region (i.e., CA1), may have the same underlying mechanism; and (3) computational cost.

### Bayesian inference for fast ripple parameters

Bayesian inference was used to estimate the probability distributions of the means and standard deviations of fast ripple parameters (i.e., peak-to-peak amplitude, power frequency, power, and duration) [41–43]. The a priori probability (P(θ)) of these fast ripple parameters means was modeled as follows:

1 $$ P\left( \mu \right) \sim N\left( {\underline {x} ,2s} \right) $$

$\underline{x}$ is the pooled empirical mean, and $s$ is the pooled empirical standard deviation. Pooled empirical standard deviation was doubled to prevent generating bias given our limited empirical data.

To model standard deviation a priori probability of the fast ripple parameters, we chose a uniform probability distribution delimited by the possible ranges of each fast ripple parameter:

2 $$ P\left( \sigma \right) \sim U\left( {min, max} \right) $$

Ranges for each variable were as follows: peak-to-peak amplitude from 1 to 150 μV; power frequency from 250 to 600 Hz; duration from 1 to 500 ms; and power from 1 to 150 $\frac{\mu {V}^{2}}{Hz} .$ Finally, the likelihood of each fast ripple parameter (*P*(X|θ)) was modeled as a T distribution with the mean and standard deviation observed in each region of the trisynaptic circuit. Degrees of freedom (ν) of the T distribution followed an a priori exponential distribution with 30 mean, which favors the regions with normally distributed data over the tails. All the main procedures followed the BEST method [43].

3 $$ P(X|\theta ) \sim T\left( {\nu ,\mu_{region} ,\sigma_{region} } \right) $$

To obtain the posterior approximate (P(θ|X)) for each fast ripple parameter mean-standard deviation pair, a Monte-Carlo Markov chain-sampling method in the PyMC3 library was used.

Value of this approach offers a variety of benefits; first, we are estimating rather than testing, because a full probabilistic description of the credibility area in which those parameters can be presented was generated; and secondly, Bayesian estimation accounts inherently for uncertainty, related to the lack of knowledge of the parameters, the stochasticity of the system, and the number of our samples.

### Multiunit activity analysis during fast ripple events

We followed the protocol published in [44], in which the signals are > 300 Hz high pass filtered using forward–backward zero-phase FIR filters of order 512, Z-transformed, and separated from the Local Field Potential (LFP) by applying a threshold of two Z-scores. To construct the probability distributions of the multiunit activity per region, samples of 400 ms were taken centered on the maximum peak-to-peak amplitude of the fast ripples, and the number of spikes was counted in 20-ms bins. The probability was calculated as dividing the total count per bin by the number of observations times the bin width.

### Wavelet coherence analysis of fast ripple events

Analysis of the frequency relationships among the different regions of the trisynaptic circuit was performed by calculating the wavelet coherence for the theta (4–7 Hz) and gamma (30–90 Hz) bands. Samples of 400 ms were taken centered on the maximum peak-to-peak amplitude of the fast ripples to perform paired comparisons of the mean coherence between the resulting 200-ms before and after segments.

### Granger causality test on fast ripple events

We modeled the causal relationships between pairs assuming EEG signals to be linear stochastic processes of stationary covariance, as defined in [45–47]. The following causal relations were proposed: *if the signal in a region X contributed to the estimation of the signal in region Y, then the past values of X should contain information that helps predict Y better than the past values of Y alone*. The analysis was performed based on [48–50], in which Granger causality is described as a bivariate autoregressive model. The number of lags in the model between pairs of EEG signals was determined using the Bayesian information criterion [51].

Samples of 200 ms before and after the fast ripples event were used to analyze the causal dynamics involved in the development of fast ripple activity. Pairwise analysis among the three registered regions of the trisynaptic circuit (DG, CA3, CA1) was performed. We obtained a 3 × 3 p-value matrix of the causal interactions between pairs before and after the fast ripples for each event (with an empty diagonal because the causality of the region with itself was not analyzed). The autocorrelation vector was used to verify the stationary covariance of the signals (i.e., the first two statistical moments of the signal should not vary with time). Two out of the 42 fast ripple samples did not accomplish the constraint and were not analyzed.

### Causal meta-analysis

To create a representative pre- and post-fast ripple causal network, each causal interaction per fast ripple was considered a sub hypothesis of the same null hypothesis (i.e., zero causality), and a statistical meta-analysis was applied. First, the Bonferroni correction method was used to adjust multiple comparisons. Second, the general probability per connection was obtained using the normal curve method [52]:

4 $$ Z = \frac{{\underline {x} - 0.5}}{.2887 / \sqrt n } $$

$\underline{x}$ is the sample mean of the $n$, and $n$ is the causal interaction probabilities, and the proportion of area under the normal curve with the related Z-score will be the overall probability associated with the pooled causal interaction probabilities. We report only connections with an overall p-value < 0.05.

### Causality-related parameters

To analyze causal connectivity, two related parameters were measured. The *unweighted unit causal density* measures interaction and the dynamic complexity of a node in relation to the full system; high values indicate global coordination but distinct dynamics between nodes [48, 49]. The nodes with high unweighted unit causal density were considered causal hubs and calculated as the summation of significant causal interactions involving a node and normalized to the number of nodes. On the other hand, the *unweighted causal flow* is considered the difference between the out-degree and in-degree measures by node; nodes with high positive unweighted causal flow were interpreted as exerting a high causal influence on the whole system and are called causal sources, whereas nodes with a high negative unweighted causal flow are called causal sinks [48, 49].

### NeuN immunofluorescence

Sham group consists of rats with surgical procedure but without the pilocarpine injection (n = 3) was used to compare the neural loss versus a fast ripples group (n = 4). Animals in both groups were euthanized with sodium pentobarbital (200 mg pentobarbital/kg body weight) before they were intracardiac perfused with 4% paraformaldehyde and brain removed to make 30 µm tissue samples with a vibratome. Sections of the brains were incubated with the specific primary antibody for NeuN immunochemistry (70-2020, dilution 1:1000, ThermoFisher). Samples were unmasked by stirring in a 2 N HCl solution by 30 min, and then 4 washes of 10 min with Phosphate-Buffered Saline (PBS, pH 7.4) were given, then tissue samples were incubated in a blocking solution (10% normal goat serum and 0.3% Triton X-100 in PBS). To subsequently be washed with PBS and incubated with the secondary antibody (Alexa Fluor 594, dilution 1:1000, Abcam) under stirring for two hours (in dark). Finally, they were mounted on slides and covered with vecta-shield (Vector Laboratories, Inc. USA) to preserve the fluorescence before covering them with a coverslip. Then the samples were examined in a fluorescence microscope with a 40× objective (Olympus, U-LH100HG, Japan) to take photos and analyze them with ImageJ 1.48 software (NIH, Bethesda, USA), cellular count was made by taking three images to complete a total area of 0.75 mm^2^ for every region (i.e., DG, CA3, CA1) analyzed for each animal, results are reported as the mean and standard deviation.

### Statistical analysis

Variables are presented as the means and standard deviations or medians and interquartile ranges, normality was assessed using a Q–Q plot. If Q–Q plots suggested a lack of normality, the Lilliefors test was used to test it. Wilcoxon and Kruskal–Wallis tests were used for nonparametric data. Statistical significance was defined as obtaining *p-*values < 0.05.

## Results

**Fig. 1:**
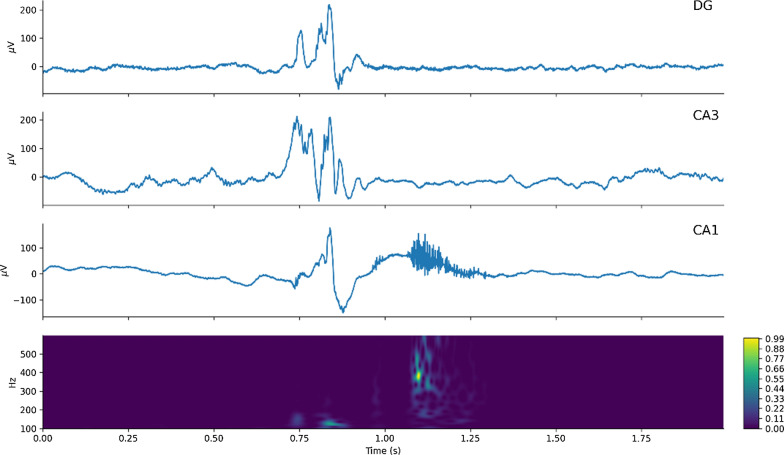
Representative fast ripple event recorded in CA1 with simultaneous raw intracranial EEG recordings from CA3 and DG. The last row shows a continuous morlet wavelet transform of the signal in CA1, in the 100–600 Hz band

To confirm that all fast ripple candidates were effectively in the 250–600 Hz bandwidth, a continuous wavelet transform (CWT) was applied to evaluate that the spectrum temporally corresponded with the high-frequency activity in the EEG recorded and that there were no harmonics of 60 Hz (Fig. 1).

**Fig. 2:**
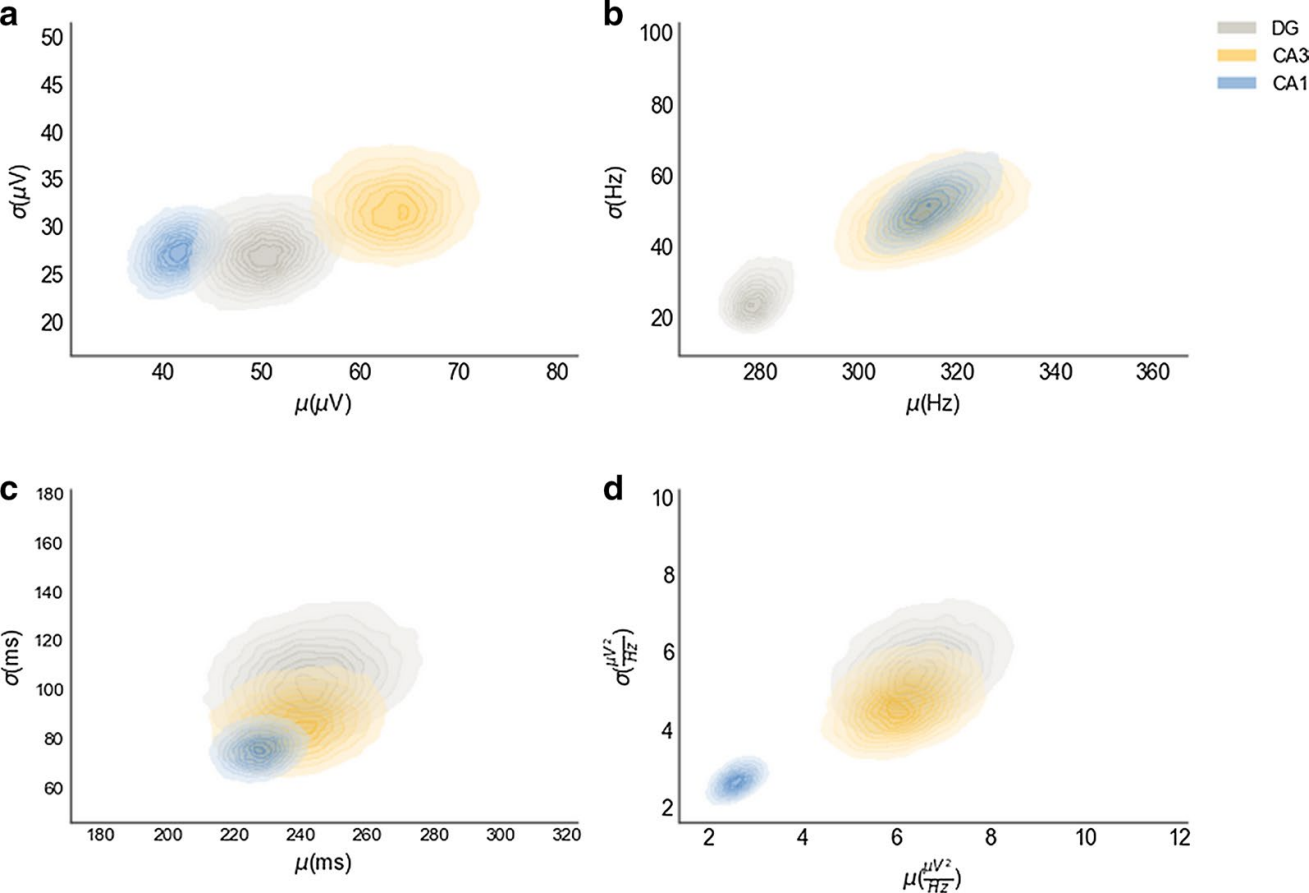
Bayesian inference of the fast ripple parameters. The surfaces in the plots describe joint posterior probability distribution of (μ) means and (σ) standard deviations in each trisynaptic circuit region of **a** peak-to-peak amplitude, **b** power frequency, **c** duration and **d** power. The inner surface of each credibility region represents the maximum a posteriori Probability (MAP) estimate value, n = 336 fast ripple events observed in 6 animals

To describe the fast ripple events observed in the present experiments, we characterized the events probabilistically (Fig. 2) considering four parameters: peak-to-peak amplitude, power frequency, duration, and power. For the peak-to-peak amplitude (Fig. 2a), registered fast ripples were in the range of 8 and 105 μV, the maximum a posteriori probability (MAP) (area which maximizes the probability, in this case, closer to the inner circle center of each credibility region) of peak-to-peak amplitude, which was 42 ± 28 μV (mean ± sd) in CA1 and was the lower ranges of estimated amplitudes. However, there was much more certainty (the area of the credibility region is smaller) in this parameter for CA1 compared to the DG and CA3, which had estimated MAP of 50 ± 26 μV and 64 ± 33 μV, respectively (Fig. 2a). The CA3 region exhibited the highest peak-to-peak estimated amplitudes in a consistent manner.

Power frequency (Fig. 2b) of fast ripple events was between 255 and 385 Hz, and the CA1 and CA3 regions generated almost identical probability distributions with estimated MAP of 318 ± 56 Hz and 315 ± 49 Hz, respectively (Fig. 2b). However, greater certainty was observed in the CA1 region. Lower power frequency events were found in the DG, with an estimated MAP of 278 ± 22 Hz. The means and the standard deviations were more consistent in this area. Therefore, we observed greater certainty in this parameter compared to CA1 and CA3. FR duration was the most homogeneous parameter among all regions because the three resulting probability distributions overlapped, even when there were differences in the certainty on the duration, which was between 138 and 400 ms (Fig. 2c).

Regarding the power, fast ripple events had all of their possible values within 0.01 and 15 $\frac{\mu {V}^{2}}{Hz}$, with CA1 concentrating less power on the power frequency with a MAP of 2.3 ± 2.1 $\frac{\mu {V}^{2}}{Hz}$. By contrast, CA3 and the DG maintained homogeneous distributions with slight displacements and estimated MAP of 6.3 ± 5.9 and 6 ± 4.8 $\frac{\mu {V}^{2}}{Hz}$, respectively (Fig. 2d).

**Fig. 3:**
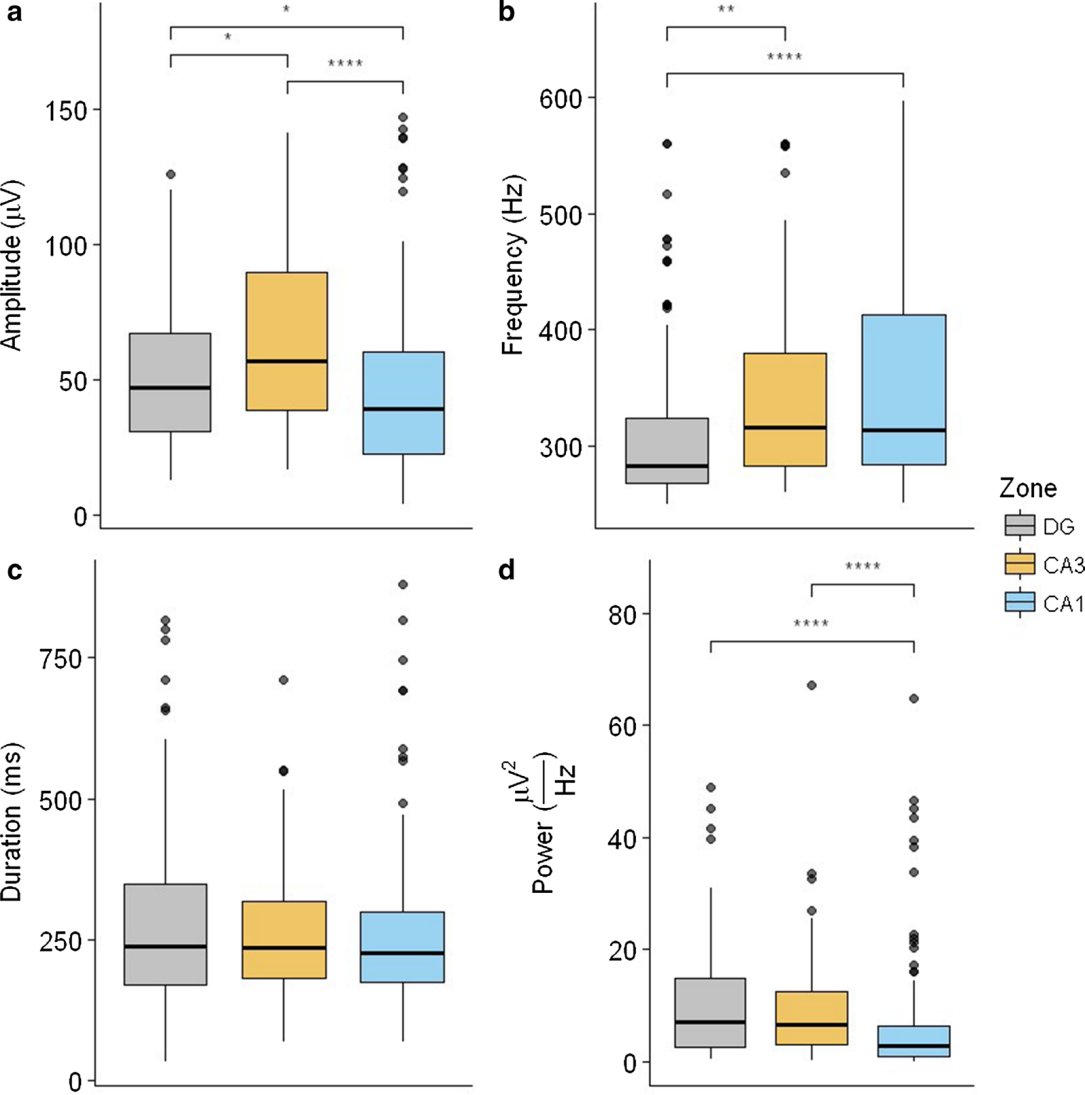
Statistical comparison of the fast ripple parameters. The box plots describe the distribution of the parameters in our data per trisynaptic circuit region of **a** peak-to-peak amplitude, **b** power frequency, **c** duration and **d** power. Kruskal–Wallis test with a Wilcoxon test for multiple comparisons was used (*p < .05, **p < .01, ***p < .001, ****p < .0001), n = 336 fast ripple events observed in 6 animals

To measure differences in the fast ripple parameters, a conventional comparison approach was applied. Regarding peak-to-peak amplitude (Fig. 3a), differences between CA1 and CA3 (p = 2.13 × 10^–6^, Wilcoxon test), between CA1 and the DG (p = 1.98 × 10^–2^, Wilcoxon test) and between CA3 and the DG (p = 2.13 × 10^–2^, Wilcoxon test) were observed. The results for power frequency (Fig. 3b) showed differences between CA3 and the DG (p = 2.19 × 10^–3^, Wilcoxon test) and between CA1 and the DG (p = 5.63 × 10^–5^, Wilcoxon test). For duration (Fig. 3c), there were no significant differences. Power (Fig. 3d) differences between the DG and CA1 (3.16 × 10^–6^, Wilcoxon test) and between CA3 and CA1 (p = 3.36 × 10^–6^, Wilcoxon test) were observed, which confirmed that the differences were significant in cases where the credibility regions did not overlap in relation to the MAP in Fig. 2.

**Fig. 4:**
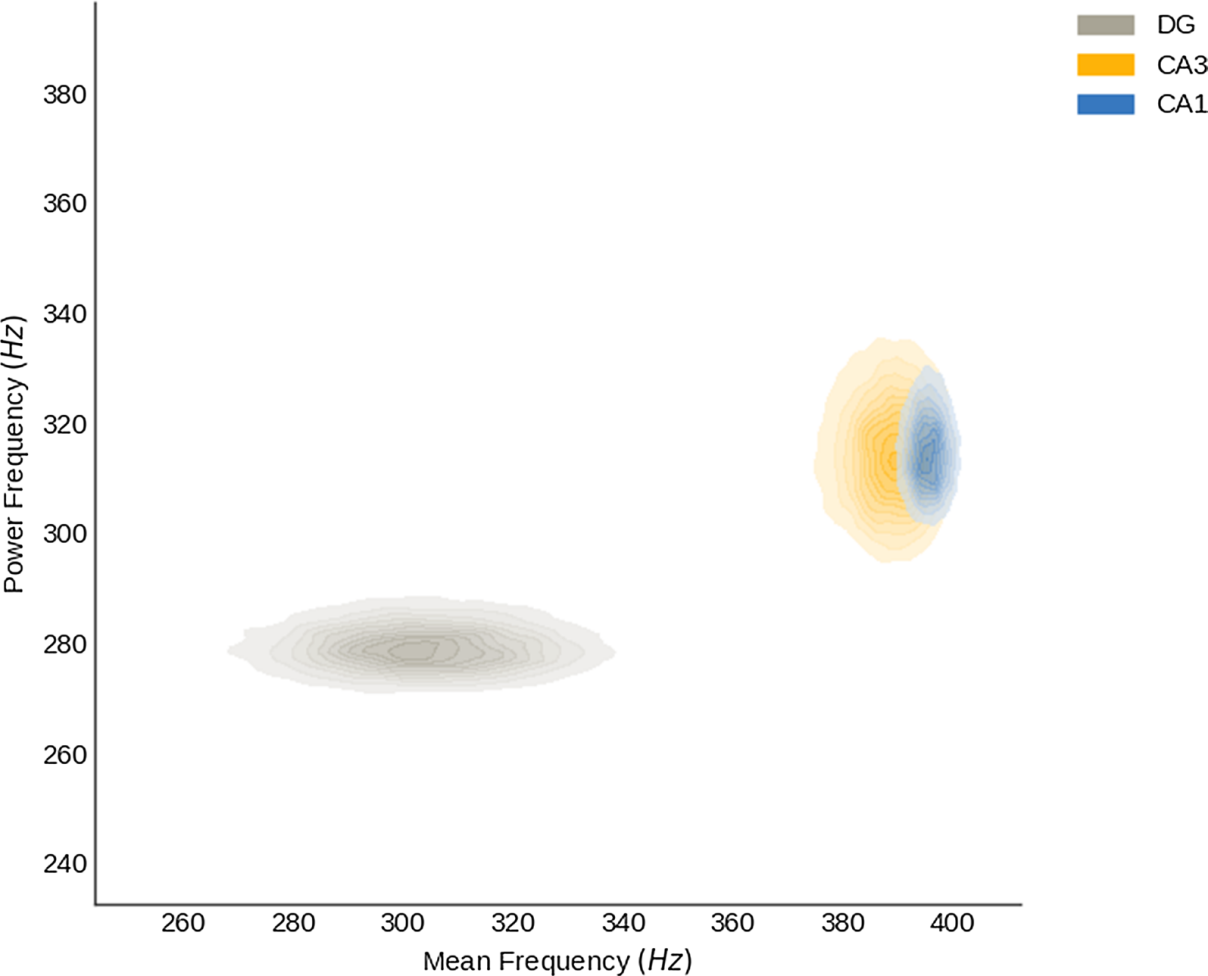
Spectral composition of fast ripple events as a function of the posterior distributions of means of power frequency and mean frequency per region

To inspect the spectral differences in fast ripples between regions, the posterior probability density of means of the additional parameter/mean frequency was estimated, and then the posterior probability density of means of power frequency was plotted as a function of the latter. The spectral distribution of fast ripple events in the DG was different from that in CA3 and CA1 (Fig. 4).

**Fig. 5:**
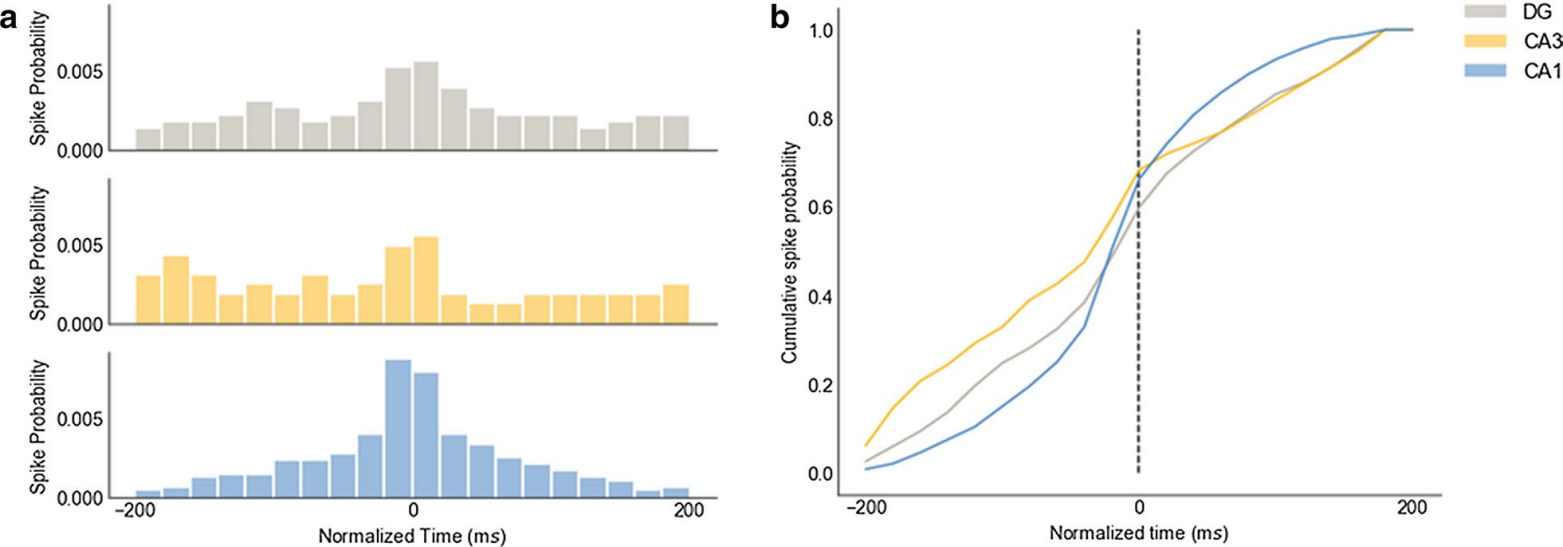
Multiunit activity at a fast ripple event. **a** Graphs show the multiunit spike probability distributions in each trisynaptic circuit region, centered at the maximum peak-to-peak amplitude of the fast ripple event. **b** Graph shows the cumulative probability distributions of (**a**). CA3 region accumulates a higher spike probability at the beginning of the event, and the CA1 region has a close to normal distribution, as expected

Probability distributions of the multiunit activity (Fig. 5a) showed synchronization phenomenon centered on the maximum peak-to-peak amplitude event (0 ms) of the fast ripples, and an expected quasi-normal distribution in CA1 was observed. The cumulative spike probability distribution (Fig. 5b) of CA1 before the maximum peak-to-peak amplitude event showed a first-order stochastic dominance over the CA3 region, which was exchanged just after the maximum amplitude event. These spike probability changes showed that CA3 was the most likely region to spike synchronously before the event, and highlight the role of CA3 in the initiation of FR events.

**Fig. 6:**
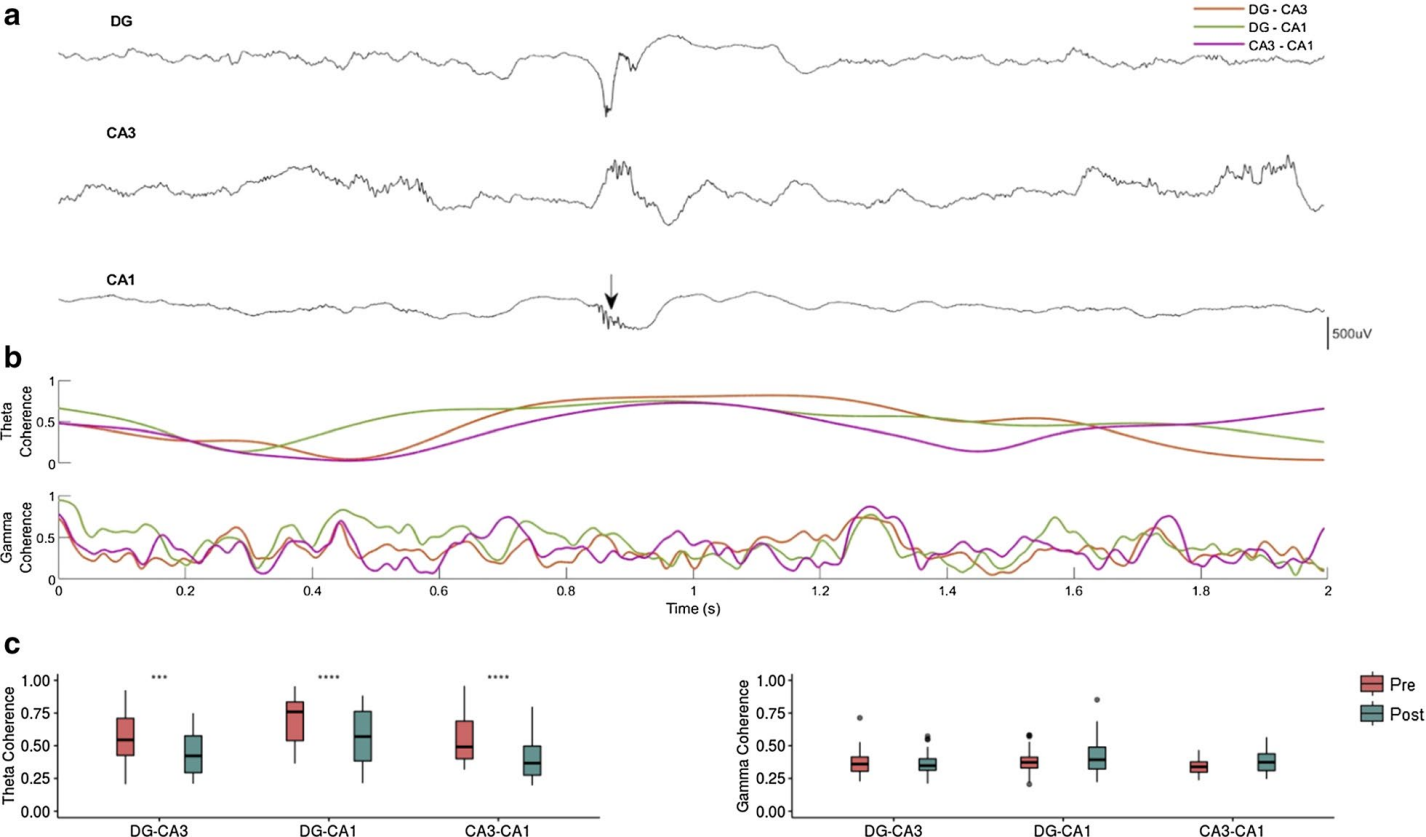
Time–frequency relationship among trisynaptic circuit regions at a fast ripple event. **a** Raw intracranial EEG recordings in each trisynaptic circuit region. Arrow is pointing to the maximum peak-to-peak amplitude of the fast ripple event recorded in CA1. **b** Wavelet coherence of the theta band (3–7 Hz) and gamma band (30–90 Hz) between regions. Note that the fast ripple is centered when theta coherence among all regions is increased. **c** Statistical comparison of the mean coherence amplitude among the trisynaptic regions 200 ms before and after the maximum fast ripple peak-to-peak amplitude in the theta band (3–7 Hz) and gamma band (30–90 Hz). Calibration bar: 500 μV. The paired Wilcoxon test per connection was used (*p < .05, **p < .01, ***p < .001, ****p < .0001)

To inspect the synchronization phenomenon needed between the analyzed regions to develop a fast ripple event, we analyzed the synchronization around two frequency bands: theta and gamma (Fig. 6a). An increase in coherence in theta band close to the center of an FR event was observed, and the coherence in the gamma band fluctuated.

There was greater coherence in the theta band among the regions before the event of maximum peak-to-peak amplitude of the fast ripples than after (Fig. 6b) and was significant for the three pairs: DG-CA3 (p = 1.4 × 10^–4^, paired Wilcoxon test), DG-CA1 (2.3 × 10^–6^, paired Wilcoxon test) and CA3-CA1 (p = 6.3 × 10^–8^, paired Wilcoxon test). These results support the theta rhythm as a facilitator of CA1 fast ripple activity. No significant differences in the gamma band were observed (Fig. 6c).

To describe the directionality of the interactions between the regions in relation to the development of a fast ripple event, we used Granger causality test 200 ms before/after each of the 40 sharp-wave associated fast ripple events. Then, to test the significance over the 40 samples, a meta-analysis was conducted calculating the overall p-value for each possible connection pair (accounting for significant and not significant interactions). All of this information is summarized in Table 1.

**Table 1:** Summary of causal interactions

Connection	Pre-FR counts	Overall p-value	Post-FR counts	Overall p-value
DG to CA3	25	0.027*	31	$2.63 \times {10}^{-8}$***
DG to CA1	9	1	17	0.983
CA3 to DG	30	$1.46\times {10}^{-5}$***	16	0.985
CA3 to CA1	34	$1.95 \times {10}^{-14}$***	36	$3.29 \times {10}^{-17}$***
CA1 to DG	12	0.999	18	0.863
CA1 to CA3	27	0.004**	30	$3.15\times {10}^{-7}$***

The number of significant causal connections of the fast ripple events was analyzed. The overall p-value was calculated using the normal curve method

**Fig. 7:**
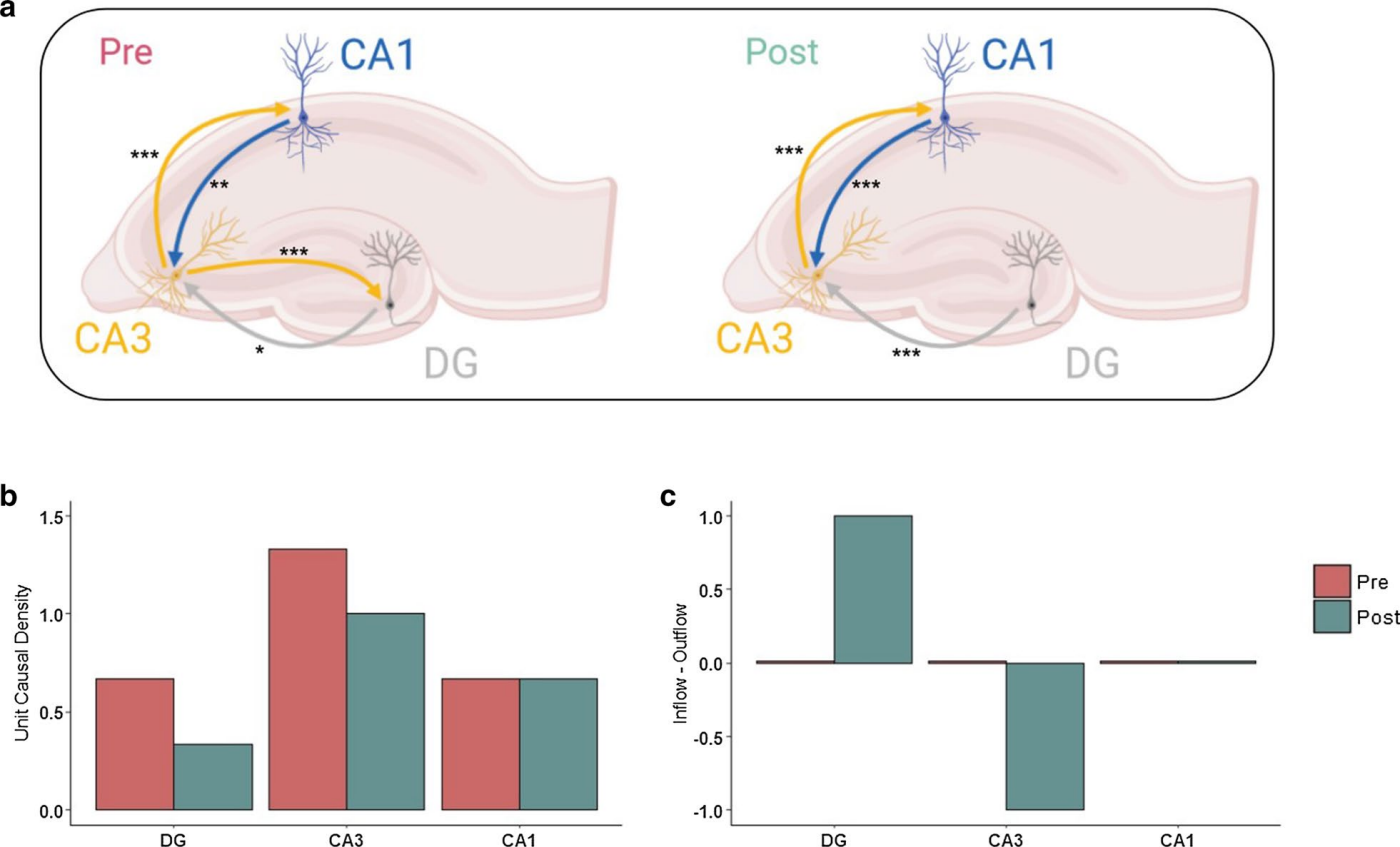
Overall pre- and post-fast ripple causal networks. **a** Schematic diagram of the resulting pre- and post-fast ripple causal network. Granger causality test was used. Only overall statistically significant connections (Table 1) are drawn (*p < .05, **p < .01, ***p < .001, ****p < .0001). In the precircuit, note the relevance of CA3 back-projection in the generation of fast ripples, and the "conventional" trisynaptic circuit is re-established in the postcircuit. The image was elaborated according the results of data analysis and is our own authorship. **b** Causal unit density and **c** causal flow among the trisynaptic circuit regions. Note the changes in **b** causal density in CA3 and the *dentate gyrus* (DG) before and after the fast ripple event. CA1 maintains its causal dynamics unchanged, and CA3 is always a causal hub. Also in **c**, the undetermined causal flow preceding the fast ripple may be interpreted as an increase in entropy in the circuit due to reverberating feedback between regions. The “conventional” causal flow of the circuit after the fast ripple event is re-established with the DG as the causal source and CA3 as the causal sink

Analysis showed that in the 200 ms lapse before the fast ripple event, the CA3 back-projection to the DG was preponderant in the development of fast ripple activity compared to the resulting circuit once the fast ripple activity was finished. This resulting circuit after the fast ripple activity was similar to the “canonical” trisynaptic circuit (Fig. 7a). The unit causal density (Fig. 7b) before the fast ripple event was increased in the DG and CA3, which highlights the CA3 region as a causal hub and the DG as the interaction support region for the development of fast ripple activity. Moreover, there was no net causal flow before the fast ripple event, but the expected causal flow from the DG to CA3 was re-established after the fast ripple event (Fig. 7c).

## Discussion

Bayesian inference of fast ripple classic parameters, such as peak-to-peak amplitude, power, frequency, and duration, showed similar results to those produced in previous in vitro studies [26, 53]. However, in vivo studies found a mean amplitude of 0.5 mV in the kainic acid model in rats (range 0.2 to 1.5 mV) and patients with TLE (0.4–0.5 mV) [28, 29, 31, 33, 54–56]. These results are different from the present study, in which an amplitude range of 8–105 µV was observed. This difference is because peak-to-peak amplitudes greater than 150 μV in the analysis were eliminated (see methods section). The amplitude found in the kainic acid model was higher than the present study likely due to the use of a different model of TLE and the different number of microelectrodes. The analyzed area was larger in these studies and included different depths. However, the probability distribution of the power frequency and duration means observed in the present work (255–383 Hz and 138–400 ms, respectively) were similar to previous in vivo studies, in which ranges of 250–500 Hz and 20–500 ms were found, respectively [28, 29, 31, 33, 54–56], in addition advantage of the Bayesian method is that the probabilistic distributions generated might be helpful to scientists who want to estimate size effects of an intervention or that are looking for plausible value ranges over those parameters in a similar experimental setting.

Additionally, we inspected the spectral content of the fast ripple events, measured as the joint distribution of mean frequency and power frequency of fast ripple events, this analysis showed differences between DG and the other two regions analyzed, CA3 and CA1.If power and mean frequency of the power spectral density are nearly the same, it is more likely that the activity was a result of synchronous in-phase bursting, because the majority of the spectrum is contained by the power frequency, and other frequencies do not contribute in a significant way in such spectrum; in the other case, if other frequencies than the power frequency are important in the spectral configuration, that shall be sawed as a shift between the mean and the power frequency, and that spectral configuration is more likely to correspond to emergent, out of phase firing [22].

In the case of this work, due to the slight shift between the MAP estimations of power frequency (280 Hz) and mean frequency (300 Hz) in the DG region, it is more likely that fast ripple activity in this region was produced by synchronous in-phase cellular bursting, possibly due to loss of interneuronal activity drive in DG and subsequent synchronous firing of granular cells [57–59], as we observed it in the histological results in which a decrease in the number of neurons in DG was found (Additional file 1: Figure S1); in contrast, CA3 and CA1 had a drastic shift between power frequency (315 Hz and 318 Hz respectively) and mean frequency (385 Hz and 398 Hz respectively) according their MAP estimations, describing a more diverse spectral configuration, probable because fast ripple events in those areas, had other relevant higher frequencies in its spectral composition than the power frequency. That spectral composition is more likely under asynchronous firing schema; this could be explained by evidence in which an emergent out of phase firing in pyramidal cells of CA3 and CA1 has been seen in relation with the fast ripple generation process [22, 53].

Besides the distribution of the parameters and its differences between regions, this work gathered evidence to help to understand the mechanism of fast ripples generation in vivo. Our multiunit activity analysis indicated that multiunit activity during the development of the fast ripple activity began with a higher probability in the CA3 region. Similar observations were found in the transition of ripple activity from CA3 to CA1 [60], and these data are consistent with in vitro studies of fast ripples [53] and with previous results [14, 23]. Presumably, this phenomenon is observable when there is no correct inhibitory control over the CA3 region due to neuronal loss, primarily interneurons [61] and mossy cells at the polymorphic layer (hilus) of the DG [59, 62, 63].

This imbalance seems enhanced by the plastic properties of the hippocampus, first by the outbreak of new axonal connections of mossy fibers towards CA3 apical dendrites [56, 64] and mossy cells [59], and second, likely due to a reorganization of the network. Studies have shown that this epilepsy-related network reorganization included the death of mossy cells and reactive neurogenesis of granular cells [65, 66], which creates pathological motifs between granular cells [15, 67]. Those motifs have shown hyperexcitability [15], which results in an improper excitation of basket cells [68] and thus an incorrect inhibitory control in DG. This reorganization has been shown to be consolidated via long-term potentiation [69] in which the interaction between CA3 pyramidal cells, granular cells, and surviving mossy cells seems to be fundamental [15, 66, 70], which also agrees with the pathological activity of the network found in vitro [53].

Regarding the G-causality analysis, the two “non-canonical” trisynaptic back-projections caught our attention (CA1 to CA3 and CA3 to DG), in the case of the CA1 to CA3 connection, a recent work has physically mapped this connection, using multiple retrograde viral tracing approaches, several and significant “non-canonical” synaptic inputs to dorsal hippocampal CA3 from ventral CA1, perirhinal cortex, and the subicular complex have been found [71]. Functional role of this connection remains unclear but it could be related with the auto-associative function of CA3, even though, some of this “non-canonical” inputs appear to have a role in pathological conditions such as epilepsy, particularly, disrupting the local network rhythms and modulating the spike timing by a theta wave “reverse flow”, boosting the propagation of epileptiform events [71–73].

In the case of the CA3 to DG connection, we think that the CA3-mossy cells axis could be mediating the activity of this connection because its coherence increases in the theta band before the fast ripple event for all pairs of regions analyzed (suggesting a primordial generator), and one of the main theta oscillators in the hippocampus is mediated by the CA3—mossy cells axis, that should be suppressed by DG interneuronal activity in a healthy brain [59, 74–77]. In contrast, the CA3—mossy cell axis has shown exacerbated activity that is long-term potentiated by theta rhythm in pathological conditions [78]. Moreover, recently it has been observed that theta activity was fundamental for development and spread of epileptic activity [44], being this coupling between slow oscillations and fast ripples found in patients with epileptic spasms [37, 79] and TLE patients with memory deficits [80–84].

In fact, the cognitive impact of TLE seems to be related with the imbalance and reorganization of the hippocampal network discussed above, in particular with the function of the CA3-DG back-projection (the only difference between the causal connections before and after the fast ripple event), that has gained relevance in recent years in both healthy and pathological conditions [58, 59]. In pathological conditions, the hyperexcitability of the DG related to the recurrent excitation of CA3-DG feedback mediated by mossy cells appears to decrease the ability of the DG to filter information and separate patterns [57, 85], which directly impacts pattern processing and completion in the CA3 region [86–90], specifically, under an epileptic hippocampus with poor temporal control and firing selectivity, the mechanisms of ripple generation, in which the CA3-DG back-projection and mossy cells [91] along with a decreased inhibitory drive [92] are involved, seem also to play a fundamental role in the generation of FR events. This overlap has also been seen in the decrease of information coded in ripple activity of epileptic rats, a phenomenon described in [14], and would explain why the increase in the fast ripple/ripple ratio was related to reduced hippocampal volume and neuronal loss in patients with TLE [34].

Similar observations were reached from the graph analysis of the resulting causal network before and after the fast ripple events. First, the causal flow before the fast ripple phenomenon was equal to zero for all nodes, which describes a circuit with complete feedback, i.e., it is not possible to determine which node is having the greatest causal influence a priori (because all of the in-degrees and out-degrees for each node are equal). However, compared to the resulting circuit after the FR events, the causal flow showed a defined route in which the DG acts as a causal source and the CA3 acts as a causal sink, a description that coincides with the auto-associative role of the CA3 [8, 9, 87, 88, 90, 93, 94] and the inhibitory gating role of the DG [95–97].

Notably, the causal connections between CA3 and CA1 (and therefore the causal flow and causal density) were not altered by the fast ripple events. Similarly, the unit causal density in the CA1 region also remained unchanged, and it was higher in the DG-CA3 pair before than after the fast ripple events. High causal density values describe global coordination but different dynamics between nodes [49]. Therefore, for the generating mechanism of fast ripple events in CA1, there must be global coordination between the CA3-DG pair, but each node contributes to the phenomenon in different ways. Given these observations, the result of the loss of control in the DG-CA3 pair, caused by the back-projection between CA3 and the DG, and the fact that there were no changes in CA1 unitary causal density from before to after the fast ripple event suggest that the only change needed in CA1 to develop fast ripple activity is an intrinsic causal change, which could be the alteration of firing dynamics due to the loss of balance in excitatory-inhibitory drive in that region described in [14].

Relevance of this connection to pathological processes of the hippocampus has been demonstrated not only through mathematical inferences, such as in the present work, but also through physical measurements, such as assessment of long-term potentiation of the CA3-DG back-projection pathway, which plays an important role in the propagation of epileptic activity mediated by theta rhythm [78], and current flow in current source density analysis in brain slices, in which an important role of the CA3 back-projection to the DG in early fast ripple generation was reported in vitro [53].

## Conclusions

This work supports the evidence that CA3 initiates fast ripple activity and adds information in vivo about how the CA3-DG back-projection seems to play a fundamental role in developing this seizure-related activity. Pathological changes in this projection lead to a shift from an inhibitory to a recurrent reverberating excitatory role, which seems to be a change needed to develop and consolidate pathological activity in the hippocampus.

## Supplementary Information

**Additional file 1: Figure S1.** Representative images obtained by NeuN immunofluorescence in both sham (n = 3) and epileptic groups (n = 4) in *dentate gyrus* (DG, sham: 630 ± 31.2, epileptic: 447.5 ± 18.3, T-test *** p < 0.0001) CA3 (Sham: 330 ± 66.5, Epileptic: 343 ± 37.3), and CA1 regions (Sham: 322 ± 14.5, Epileptic: 352 ± 27.5, T-test * p < 0.01).

## Data Availability

The data used to support the findings of this study are available from the corresponding author upon request.

## Abbreviations

CWT: Continuous wavelet transform
DG: Dentate gyrus
EEG: Electroencephalogram
FR: Fast ripples
LFP: Local field potential
MAP: Maximum a posteriori probability
PBS: Phosphate-buffered saline
TLE: Temporal lobe epilepsy
