# Supplementary figures and images for: Causal relationship of CA3 back-projection to the dentate gyrus and its role in CA1 fast ripple generation
Source: BMC Neurosci. 2021 May 17;22:37. doi: 10.1186/s12868-021-00641-4 (PMC8130286; doi:10.1186/s12868-021-00641-4)

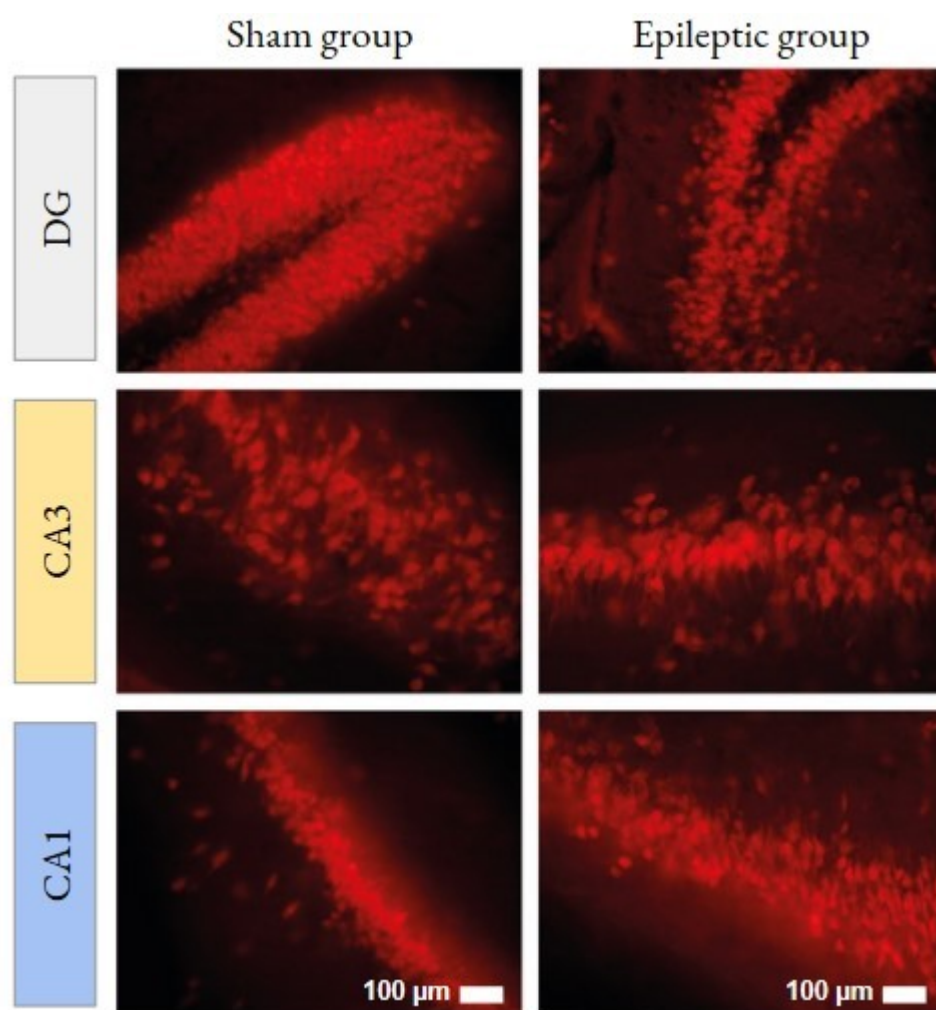

Supplement: Supplementary file 1 — Additional file 1: Figure S1. Representative images obtained by NeuN immunofluorescence in both sham (n = 3) and epileptic groups (n = 4) in dentate gyrus (DG, sham: 630 ± 31.2, epileptic: 447.5 ± 18.3, T-test *** p < 0.0001) CA3 (Sham: 330 ± 66.5, Epileptic: 343 ± 37.3), and CA1 regions (Sham: 322 ± 14.5, Epileptic: 352 ± 27.5, T-test * p < 0.01). [file 12868_2021_641_MOESM1_ESM.pdf]
